# Early Changes in Health-Related Quality of Life as a Biomarker of Survival in African Patients with HIV-Associated Kaposi Sarcoma

**DOI:** 10.3390/tropicalmed9100244

**Published:** 2024-10-17

**Authors:** Fahmida Shaik, Thomas S. Uldrick, Mikateko Mazinu, Nomonde Gwebushe, Anisa Mosam

**Affiliations:** 1 Department of Dermatology, University of Kwa-Zulu Natal, Durban 4001, South Africa; mosama@ukzn.ac.za; 2 SAMRC Clinician Researcher Development Scholarship PhD Programme, Tygerberg, Cape Town 7505, South Africa; 3 Fred Hutchinson Cancer Center, Seattle, WA 98109, USA; tuldrick@fredhutch.org; 4 Biostatistics Research Unit, South African Medical Research Council, Tygerberg, Cape Town 7505, South Africa; mikateko.mazinu@mrc.ac.za (M.M.); nomonde.gwebushe@mrc.ac.za (N.G.); 5 Inkosi Albert Luthuli Central Hospital, Cato Manor, Durban 4091, South Africa

**Keywords:** Kaposi sarcoma, biomarker, quality of life, KSHV

## Abstract

Sub-Saharan Africa bears the largest public health burden of Kaposi sarcoma (KS), a leading cause of cancer mortality. Quality of life (QOL) assessments in cancer patients can provide information on prognosis beyond traditional biomarkers or biological measures. The prognostic value of QOL measures in patients with HIV-KS was evaluated. Prognostic associations of baseline QOL scores (by quartiles or thresholds for clinical importance) and changes in QOL scores (using minimum important difference) over the first 3 months of therapy were evaluated in 112 participants with HIV-KS randomised to receive ART, with or without chemotherapy. Cox’s regression analysis assessed the prognostic contribution of QOL scores from the EORTC QLQ-C30 questionnaire. Survival curves were generated using the Kaplan–Meier method. Baseline QOL scores did not predict overall survival. The change in the 3-month QOL scores for the global health scale, fatigue, and pain domains was prognostic; the hazard ratios were 3.88 (95% CI 1.32–11.38, *p* = 0.01), 3.72 (95% CI 1.61–8.62, *p* = 0.00) and 5.96 (95% CI 2.46–14.43, *p* = 0.00), respectively. QOL assessments can provide useful prognostic information in patients with HIV-KS. Patients lacking meaningful improvement early into treatment represent a population at high risk of death.

## 1. Introduction

Kaposi sarcoma (KS) remains a leading cause of cancer incidence and mortality in parts of sub-Saharan Africa [1]. It is the most common cancer among HIV-infected individuals, despite the advances in HIV care [2,3]. Global progress against HIV remains slow, with approximately 1.5 million new HIV infections and 650,000 AIDS-related deaths in 2021 [4]. KS will, therefore, continue to be a public health concern.

The causative organism is Kaposi’s sarcoma-associated herpes virus and KS can have a spectrum of clinical presentations ranging from indolent disease with focal lesions to extensive or systemic disease with severe morbidity and mortality [5]. The presence of KS skin lesions is also associated with increased HIV-related stigmatisation [6]. The disease can be controlled, and quality of life (QOL) can improve with antiretroviral therapy (ART), and an addition of systemic therapy can be made for extensive disease [7].

Chemotherapy is commonly used to treat KS and can have significant palliative benefits, but it can also result in cumulative toxicities [8]. Optimal treatment should aim to reduce disease progression and achieve a balance between prolongation of life and satisfactory QOL. QOL measurements provide the patient’s perception of their disease status and care. QOL measures, using mainly the EORTC QLQ-C30, can also be strong prognostic factors for survival in patients with a variety of both localised and metastatic cancers (reviewed by Gotay et al., Montazeri et al., Efficace et al. and Mierzynska et al.), with baseline/pre-treatment global health status (GHS) and physical functioning being the most frequently reported prognostic indicators [9,10,11,12].

A recent multi-centre study by Eichler et al., with 1102 sarcoma patients, found that in addition to baseline GHS, physical functioning, fatigue, pain and loss of appetite, a novel and validated summary score had prognostic significance, with GHS and this summary score having the greatest effect [13]. GHS has been considered a good indicator of overall QOL; however, the summary score was found to be more meaningful and reliable [14].

Before the availability of ART to treat HIV and promote immune reconstitution, evaluation of QOL using the functional living index—cancer (FLI-C) tool supplemented by a KS-specific module showed that baseline QOL scores predicted overall survival in patients with HIV-KS and deteriorated over time with both chemotherapy and radiotherapy [15]. However, with the availability of ART, we have previously shown that improvement in QOL can be associated with KS treatment and tumour regression [7]. In this study, both baseline and changes in QOL measures were evaluated as predictors of survival in HIV-associated KS patients receiving ART. We hypothesised that scores indicating inferior GHS, inferior physical functioning, and increased pain would be significantly associated with inferior survival.

## 2. Methods

This prospective, randomised, controlled trial KAART (NCT00380770; clinicaltrials.gov), conducted between 2003 and 2009, consisted of 112 treatment-naïve adult participants (median age 33 years) with histologically diagnosed HIV-associated KS. Visceral disease was present in 53%, and 89% had a high tumour burden (T1). Patients randomised to receive ART (53%), or ART and chemotherapy (47%), were followed for 12 months. Details of the trial, study design, participants, clinical outcomes and QOL findings (changes over time, comparison between study arms and associations with tumour response or clinical parameters) were reported previously [7,16]. Participation required written informed consent. This study was approved by the University of KwaZulu Natal Biomedical Research Ethics Committee (BE 442/16).

### 2.1. Assessments

QOL data using the validated cancer-specific EORTC QLQ-C30 questionnaire and information on survival were utilised [17]. The questionnaire was also translated into isiZulu as 98.21% of participants were Black African. QOL data were measured prospectively in all patients at baseline and 3 times monthly until trial completion (12 months). The self-reported, 30-item questionnaire scores QOL domains from 0 to 100. It has multi-item scales: a global health scale (GHS), 6 single-item measures, 5 functional domains (physical, role, emotional, cognitive and social) and 3 symptom domains (fatigue, nausea/vomiting and pain). High scores indicate better QOL for the functional domains and GHS and higher symptom burden for the symptom domains [17,18]. The summary score is calculated as the mean of the combined scales (excluding GHS and financial impact), with a higher score indicating a better QOL [14].

A change in any of the QOL measures of 10 points or more is considered clinically meaningful and regarded as a significant change. This may represent an improvement in the patient’s functioning and symptoms, referred to as minimum important difference (MID) [18,19,20]. Giesinger et al. have also recently established thresholds for clinical importance (TCI) to identify patients with clinically important restrictions at a single time point. Absolute QOL scores below the TCI for functional domains, or above the TCI for symptom domains, indicate clinically relevant problems or symptoms. There are no validated thresholds for summary score and GHS [21].

For the present analysis, we evaluated the associations of baseline QOL scores and changes in QOL scores over the first 3 months of therapy on overall survival in the KAART study. The QOL domains analysed for prognostic value included GHS, the novel summary score, fatigue, pain, physical, role, emotional and social functioning scales. Other domains lacked variability and were excluded from further analysis. The specific associations evaluated included the following: baseline QOL scores, which were investigated as a predictor of survival; survival was compared in those with clinically important problems for baseline functional and symptom domains versus those without (using TCI levels); and survival was also compared in those who demonstrated a minimum important difference in QOL scores from baseline to month 3 and those who did not.

### 2.2. Statistical Considerations

Baseline GHS, summary score, and QOL domains were described using frequencies and percentages for categorical data, while median and interquartile range were used for continuous variables. Scores for each outcome were categorised into quartiles. Baseline analyses included prognostic value of scores above vs. below a TCI. Cox’s regression analysis was performed to assess whether any of the QOL scores contributed independently to the length of survival. The survival time for each patient was the time from baseline to 12-month follow-up or the date of death. Finally, the relationship between change in QOL scores and survival was assessed. The change in QOL scores was calculated by subtracting the baseline QOL scores from the 3-month QOL scores. The analyses were adjusted for the treatment arms and the presence of visceral KS to control for potential confounding effects. Survival curves were generated based on the Cox proportional hazard model, adjusted for the treatment arm and visceral disease status. The standard significance level used in all analyses was a *p*-value <0.05. Analyses were performed using STATA version 16.

## 3. Results

After excluding 7 patients without baseline and/or follow-up QOL data, 105 (94%) questionnaires were available for analysis at baseline and 92 (82%) at month 3; 11 patients died, and 4 were lost to follow-up.

### 3.1. Baseline QOL and Survival

Baseline QOL was affected by HIV-associated KS. Median baseline scores (IQR) were 50 (41.67–66.67) for global health status, 75.15 (61.03–87.74) for summary score, 86.67 (86.67–93.33) for physical functioning, 66.67 (33.33–83.33) for role functioning, 75 (50–91.67) for emotional functioning, 66.67 (33.33–100) for social functioning, 44.44 (11.11–61.11) for fatigue and 50 (16.67–83.33) for pain. Baseline GHS, summary scores and functional and symptom domains showed no significant difference between the survivors and those who died (Table 1).

Validated TCI is available for the functional and symptom domains. Among functioning scales, scores below TCI were seen in 16 patients (15%) for physical functioning, 47 patients (47%) for role functioning, 44 patients (42%) for emotional functioning and 48 patients (46%) for social functioning. Among symptom scales, scores above TCI were seen in 53 patients (51%) for fatigue and 69 patients (66%) for pain.

Despite the demonstration that disease-related effects on function and symptoms were common, analyses of baseline scores by quartile or TCI demonstrated that baseline measures of QOL did not predict overall survival (Table 1).

### 3.2. Minimum Important Difference (MID) at 3-Months and Survival

Month 3 QOL scores were considered clinically improved if there was an increase of 10 or more when compared for the GHS and the functional domains, and a decrease of 10 or more for the symptom domains. A clinically meaningful improvement in QOL in each domain was observed in patients who survived for 12 months versus those who died. At month 3, 35% of patients showed meaningful improvement (MID) in GHS, 30% in the summary score, 10% in physical functioning, 29% in role functioning, 32% in emotional functioning, 26% in social functioning, 62% in fatigue and 53% in pain (Table 1).

In the unadjusted analyses, GHS, pain and fatigue were significantly associated with survival (Supplementary material Table S1, Figure S1). The treatment arm and visceral KS status were included as confounders in the final models. Whilst the treatment arm had no significant independent effect on the risk of death across various QOL domains, patients with the presence of visceral KS consistently had a higher risk of death.

**Figure 1:**
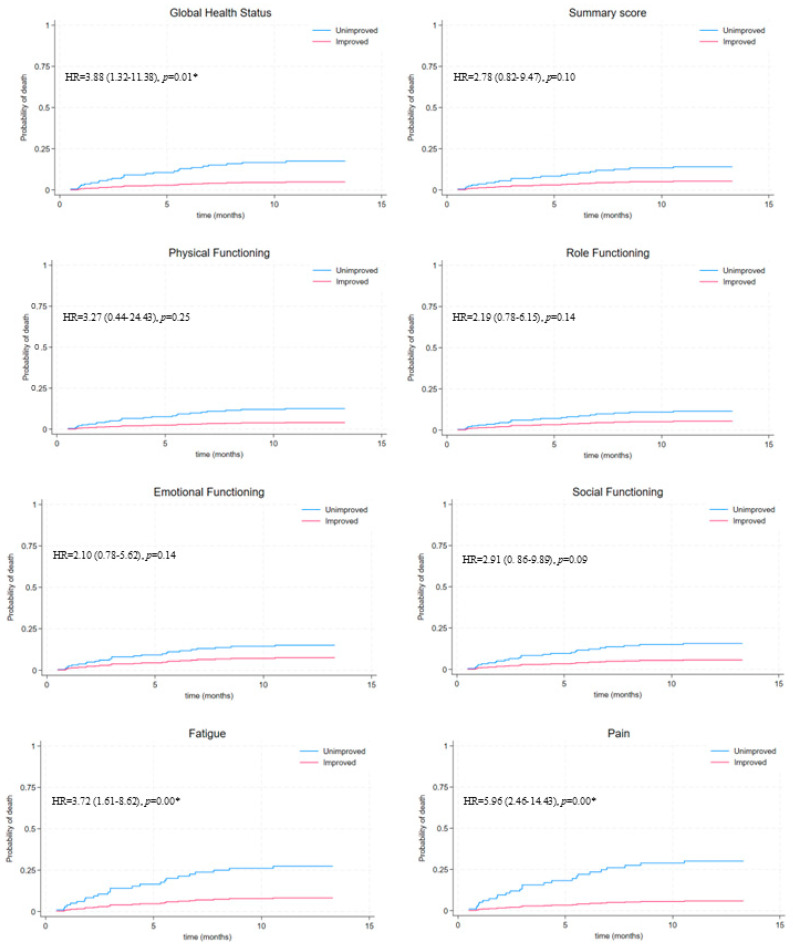
Kaplan–Meier survival curves of patients who demonstrated a minimum important difference in QOL scores from baseline to month 3 and those who did not, for each QOL domain, adjusted for treatment arm and visceral KS status. (* statistically significant at 0.05 level).

Kaplan–Meier survival curves, adjusted for the treatment arm and visceral KS status in Figure 1, demonstrate better survival in those with improved MID-QOL scores for each of the QOL domains. A statistically significant hazard ratio was still observed in those with unimproved GHS, pain and fatigue domains. The hazard ratio for the outcome (death) was 3.88 (95% CI 1.32–11.38, *p* = 0.01) greater in patients with unimproved GHS, indicating that the probability of dying is substantially higher in those without an improvement in GHS at month 3. There was a more than three times greater risk of mortality in patients with unimproved fatigue (3.72) (95% CI 1.61–8.62, *p* = 0.00) and an almost six times greater risk of mortality in patients with unimproved pain (5.96) (95% CI 2.46–14.43, *p* = 0.00) when compared to those who had improved. These significant associations between QOL domains and survival outcomes are visualised in the survival curves (Figure 1, Table 1).

## 4. Discussion

To our knowledge, this is the first study to examine the prognostic value of QOL on survival in HIV-associated KS in the ART era. Evaluating the relationship between QOL measures and survival may guide management that prolongs survival without compromising QOL. Importantly, despite the effects of HIV-associated KS on QOL in this population of treatment-naïve patients, none of the baseline QOL measures were prognostic for survival, and no significant relationship was demonstrated with baseline TCI levels and survival for all QOL domains. However, improvement in QOL over the first 3 months of treatment was prognostic, as a clinically meaningful change (MID) in GHS, pain and fatigue domains early into treatment is associated with improved survival in patients with KS.

Baseline QOL scores are representative of disease at presentation, whilst follow-up QOL scores likely indicate treatment effects. Baseline scores were high for the functional domains and low for global health status, indicating that patients perceived their overall QOL as poor. Four independent systematic reviews demonstrating the prognostic value of QOL scores in patients with mainly advanced/metastatic cancer, majority randomised-controlled trials, found that pre-treatment QOL scores provided the most reliable prognostic information. GHS and physical functioning were found to be the most frequent independent prognostic factors, and the most frequently reported prognostic symptoms were pain, fatigue and appetite loss. These findings are contrary to our study, where baseline quality of life data were not prognostic for survival, highlighting the ability of patients with advanced KS to have good long-term outcomes [9,10,11,12].

Giesinger et al. established thresholds for clinical importance to give clinically meaningful interpretation to absolute QOL scores and to identify those with functional and symptom limitations [14]. At baseline, 66% of patients indicated pain that was clinically meaningful, demonstrating that pain was a limiting factor in this cohort. Baseline functional scales (except for physical functioning) appeared to be clinically problematic in many patients. There was, however, no correlation between baseline TCI levels and survival for all QOL domains. Only one other study by Eichler et al. evaluated the relationship between TCIs and survival. They found that those with clinically important limitations in physical functioning demonstrated the highest risk of mortality [13].

The review by Montazeri found that baseline QOL measures were prognostic in advanced and solid tumours and not in early and soft tumours. In studies where pre-treatment QOL was not prognostic in cancer patients, changes in QOL scores were usually prognostic for survival. In patients with advanced breast cancer, changes in overall and physical QOL were prognostic indicators. In patients with advanced head and neck cancer, the domains fatigue and pain bordered significance [10]. In our cohort, an improvement in GHS, pain and fatigue in month 3 was predictive of survival. GHS is a simple two-question measurement in the EORTC QLC-30, where patients rate their overall health and quality of life, and the measurement is considered a good representation of overall QOL [18]. Since this measurement is patient-rated, it could be a better representation of the well-being of the patient than that which is observer-rated. A minimal clinically important difference is a difference in QOL score that patients perceive as an improvement in QOL/functions and a reduction in symptoms. QOL measures are possibly sensitive indicators of patients’ well-being, and worsening of pain and fatigue could be an early indication of progressive or advancing disease. QOL measures are often overlooked in cancer care. We, however, demonstrated the potential of QOL to improve survival outcomes in patients with HIV-associated KS.

## 5. Strengths and Limitations

KAART was a prospective, randomised, controlled trial with limited data loss and the first to examine the prognostic value of QOL on survival in HIV-associated KS in the ART era. It could be argued that the RCT, with its rigorous selection criteria, may lack generalisability, and the results are also not indicative of non-treatment-naïve patients. The lack of longer survival follow-up is a limitation. Although this study was conducted a while ago, the findings remain highly relevant, especially for ART-naïve patients, as death within the first year of KS treatment remains an important public health issue.

## 6. Conclusions

The emphasis in cancer management has always been on survival, and QOL measures were perceived as soft outcomes. Assessing QOL is crucial to cancer care and research as it provides added value to the usual clinical endpoints [22]. QOL assessments within clinical trials for HIV-associated KS patients aid in the interpretation of treatment effects and provide helpful information for healthcare providers. This study demonstrates that baseline QOL in treatment-naïve patients with advanced KS is not prognostic. At the same time, lack of meaningful improvement over the first 3 months of treatment is associated with inferior survival. HIV-associated KS patients with a disease affecting QOL should be identified for intervention aimed at controlling KS to increase survival. In the era of effective treatments for HIV, it is crucial to integrate QOL data into KS studies and validate domains that are most informative in prognostic models. QOL data are integral in determining outcomes in patients with HIV-associated KS, and it is essential to include QOL measures into patient decision-making and management.

## Figures and Tables

**Table 1:** Association of baseline QOL, baseline thresholds for clinical importance and minimum important difference (baseline to month 3) with survival, adjusted for treatment arm and visceral KS status.

QOL Domains	Baseline QOL				Thresholds for Clinical Importance (Baseline)				Minimum Important Difference (Baseline to Month 3)			
	n	QOL Scores Quartiles (IQR)	HR (95% CI)	*p*	Above TCI n (%)	Below TCI n (%)	HR (95% CI)	*p*	Improved n (%)	Unimproved n (%)	HR (95% CI)	*p*
**Global health score**	31	1. Qu (0–41.67)	2.48 (0.53–11.53)	0.25	No TCI available for global health score				35 (35)	65 (65)	3.88 (1.32–11.38)	0.01 *
	33	2. Qu (>41.67–50)	1.11 (0.22–5.59)	0.90								
	22	3. Qu (>50–66.67)	2.21 (0.45–10.98)	0.33								
	14	4. Qu (>66.67–100)	ref.									
**Summary score**	25	1. Qu (17.91–61.03)	3.00 (0.60–14.88)	0.18	No TCI available for summary score				29 (30)	69 (70)	2.78 (0.82–9.47)	0.10
	24	2. Qu (>61.03–75.15)	3.12 (0.62–15.56)	0.17								
	25	3. Qu (>71.15–87.74)	3.26 (0.68–15.72)	0.14								
	24	4. Qu (>87.74–100)	ref.									
**Physical functioning**	59	1. Qu (66.67–86.67)	2.49 (0.72–8.64)	0.15	89 (85)	16 (15)	1.98 (0.77–5.12)	0.16	11 (10)	94 (90)	3.27 (0.44–24.43)	0.25
	20	2. Qu (>86.67–93.33)	1.99 (0.47–8.47)	0.35								
	26	3. Qu (>93.33–100)	ref.									
**Role functioning**	36	1. Qu (0–33.33)	1.45 (0.38–5.57)	0.59	53 (53)	47 (47)	1.39 (0.59–3.29)	0.45	29 (29)	71 (71)	2.19 (0.78–6.15)	0.14
	34	2. Qu (>33.33–66.67)	2.43 (0.66–8.98)	0.18								
	10	3. Qu (>66.67–83.33)	0.61 (0.06–6.07)	0.67								
	20	4. Qu (>83.33–100)	ref.									
**Emotional functioning**	29	1. Qu (0–50)	0.91 (0.26–3.25)	0.89	61 (58)	44 (42)	1.31 (0.59–2.90)	0.51	34 (32)	71 (68)	2.10 (0.78–5.62)	0.14
	30	2. Qu (>50–75)	1.16 (0.35–3.85)	0.81								
	29	3. Qu (>75–91.67)	0.67 (0.18–2.49)	0.55								
	17	4. Qu (>91.67–100)	ref.									
**Social functioning**	35	1. Qu (0–33.33)	2.12 (0.89–5.05)	0.09	57 (54)	48 (46)	1.80 (0.81–3.98)	0.15	27 (26)	78 (74)	2.91 (0.86–9.89)	0.09
	38	2. Qu (>33.33–83.33)	0.83 (0.25–2.69)	0.75								
	32	3. Qu (>83.33–100)	ref.									
**Fatigue**	31	1. Qu (0–11.11)	0.69 (0.23–2.05)	0.50	53 (51)	51 (49)	0.97 (0.44–2.17)	0.94	53 (62)	33 (38)	3.72 (1.61–8.62)	0.00 *
	32	2. Qu (>11.11–44.44)	1.01 (0.37–2.71)	0.99								
	15	3. Qu (>44.44–61.11)	0.44 (0.09–2.14)	0.31								
	26	4. Qu (>61.11–100)	ref.									
**Pain**	36	1. Qu (0–16.67)	1.18 (0.38–3.72)	0.78	69 (66)	36 (34)	1.18 (0.51–2.72)	0.70	46 (53)	40 (47)	5.96 (2.46–14.43)	0.00 *
	30	2. Qu (>16.67–50)	0.90 (0.27–3.02)	0.87								
	20	3. Qu (>50–83.33)	1.14 (0.32–4.12)	0.84								
	19	4. Qu (>83.33–100)	ref.									

QOL: quality of life, HR: hazard ratio, IQR: interquartile range, CI: confidence interval, TCI: thresholds for clinical importance, MID: minimum important difference, ref: reference, * statistically significant at 0.05 level. Physical and social functioning are categorised into tertiles due to a lack of data variability. QOL scores below the TCI for functional domains or above the TCI for symptom domains indicate clinically relevant problems or symptoms. An increase in the QOL score of 10 points or more for GHS and the functional domains and less than 10 or more for the symptom domains is considered clinically meaningful.

## Data Availability

The original contributions presented in the study are included in the article/Supplementary Material, further inquiries can be directed to the corresponding author.

## Supplementary Materials

The following supporting information can be downloaded at: https://www.mdpi.com/article/10.3390/tropicalmed9100244/s1, Figure S1: Kaplan Meier survival curves between patients who demonstrated a minimum important difference in QOL scores from baseline to month 3 and those who did not, for each QOL domain, unadjusted for treatment arm nor KS visceral status; Table S1: Association of baseline QOL, baseline thresholds for clinical importance and minimum important difference (baseline to month 3) with survival, unadjusted for treatment arm nor visceral KS status..
