# Supplementary material for: Early Changes in Health-Related Quality of Life as a Biomarker of Survival in African Patients with HIV-Associated Kaposi Sarcoma
Source: Trop Med Infect Dis. 2024 Oct 17;9(10):244. doi: 10.3390/tropicalmed9100244 (PMC11511451; doi:10.3390/tropicalmed9100244)
Supplement: Supplementary file 1 [file tropicalmed-09-00244-s001.zip › tropicalmed-3136797-supplementary.pdf]

Supplementary Material

Supplementary Table S1. Association of baseline QOL, baseline thresholds for clinical importance and minimum important difference (baseline to month 3) with survival, unadjusted for treatment arm nor visceral KS status.

QOL Domains	Baseline QOL				Thresholds for clinical importance (baseline)				Minimum important difference (baseline to month 3)			
	**n**	**QOL scores Quartiles (IQR)**	**HR (95% CI)**	***p***	**Above TCI n (%)**	**Below TCI n (%)**	**HR (95% CI)**	***p***	**Improved n (%)**	**Unimproved n (%)**	**HR (95% CI)**	***p***
**Global health score**	31	1.Qu (0-41.67)	3.01 (0.67-13.57)	0.15	No TCI available for global health score				35 (35)	65 (65)	3.53 (1.21-10.30)	*0.02
	33	2.Qu (>41.67-50)	1.28 (0.26-6.32)	0.77								
	22	3.Qu (>50-66.67)	2.20 (0.44-10.92)	0.33								
	14	4.Qu (>66.67-100)	ref.									
**Summary score**	25	1.Qu (17.91-61.03)	2.97 (0.60-14.73)	0.18	No TCI available for summary score				29 (30)	69 (70)	3.03 (0.89-10.30)	0.08
	24	2.Qu (>61.03-75.15)	3.24 (0.65-16.07)	0.15								
	25	3.Qu (>71.15-87.74)	3.23 (0.67-15.59)	0.14								
	24	4.Qu (>87.74-100)	ref.									
**Physical functioning**	59	1.Qu (66.67-86.67)	2.79 (0.82-9.51)	0.10	89 (85)	16 (15)	2.44 (0.97-6.12)	0.06	11 (10)	94 (90)	3.37 (0.46-24.94)	0.23
	20	2.Qu (>86.67-93.33)	2.11 (0.50-8.84)	0.31								
	26	3.Qu (>93.33-100)	ref.									
**Role functioning**	36	1.Qu (0-33.33)	1.79 (0.48-6.61)	0.38	53 (53)	47 (47)	1.60 (0.70-3.64)	0.27	29 (29)	71 (71)	1.70 (0.63-4.58)	0.29
	34	2.Qu (>33.33-66.67)	2.01 (0.55-7.30)	0.29								
	10	3.Qu (>66.67-83.33)	0.62 (0.06-6.00)	0.68								

	20	4.Qu (>83.33-100)	ref.									
**Emotional functioning**	29	1.Qu (0-50)	0.86 (0.24-3.06)	0.82	61 (58)	44 (42)	1.12 (0.51-2.48)	0.77	34 (32)	71 (68)	2.33 (0.87-6.21)	0.09
	30	2.Qu (>50-75)	1.49 (0.47-4.74)	0.50								
	29	3.Qu (>75-91.67)	0.71 (0.19-2.64)	0.61								
	17	4.Qu (>91.67-100)	ref.									
**Social functioning**	35	1.Qu (0-33.33)	2.26 (0.85-6.01)	0.10	57 (54)	48 (46)	1.69 (0.77-3.72)	0.19	27 (26)	78 (74)	3.10 (0.93-10.35)	0.07
	38	2.Qu (>33.33-83.33)	1.00 (0.34-2.98)	1.00								
	32	3.Qu (>83.33-100)	ref.									
**Fatigue**	31	1.Qu (0-11.11)	0.65 (0.22-1.95)	0.44	53 (51)	51 (49)	1.0 (0.45-2.22)	1.00	53 (62)	33 (38)	3.34 (1.38-8.05)	*0.007
	32	2.Qu (>11.11-44.44)	0.95 (0.35-2.54)	0.91								
	15	3.Qu (>44.44-61.11)	0.42 (0.09-2.04)	0.28								
	26	4.Qu (>61.11-100)	ref.									
**Pain**	36	1.Qu (0-16.67)	0.91 (0.30-2.70)	0.86	69 (66)	36 (34)	1.10 (0.49-2.50)	0.82	46 (53)	40 (47)	5.46 (1.87-15.93)	*0.002
	30	2.Qu (>16.67-50)	0.69 (0.21-2.27)	0.55								
	20	3.Qu (>50-83.33)	0.86 (0.25-2.97)	0.81								
	19	4.Qu (>83.33-100)	ref.									

QOL: quality of life, HR: hazard ratio, IQR: interquartile range, CI: confidence interval, TCI: thresholds for clinical importance, MID: minimum important difference, ref: reference, * Statistically significant at 0.05 level.
Physical and social functioning are categorised into tertiles due to a lack of data variability.
QOL scores below the TCI for functional domains or above the TCI for symptom domains indicate clinically relevant problems or symptoms.
An increase in the QOL score of 10 points or more for GHS and the functional domains and less than 10 or more for the symptom domains is considered clinically meaningful.

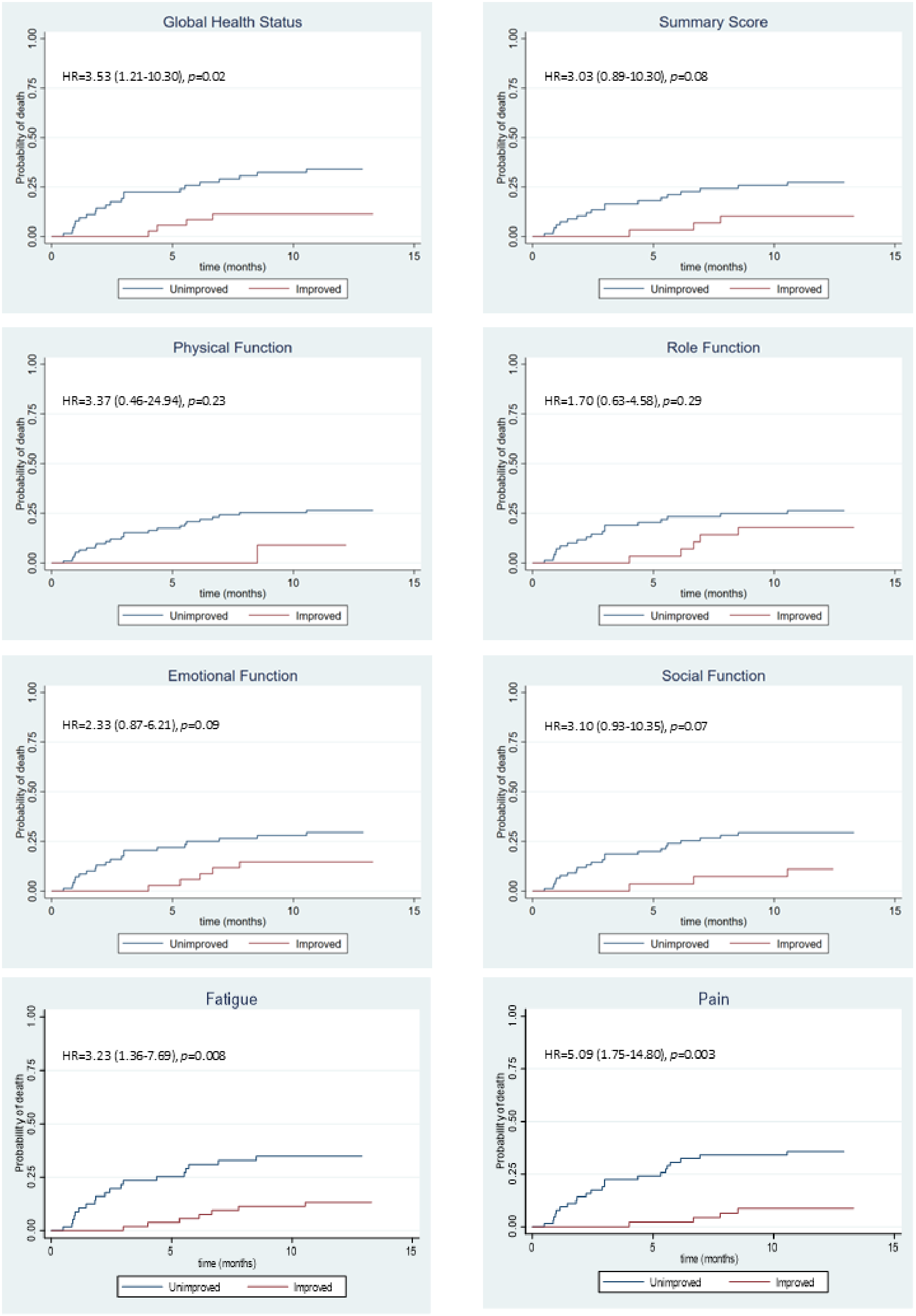

Supplementary Figure S1. Kaplan Meier survival curves between patients who demonstrated a minimum important difference in QOL scores from baseline to month 3 and those who did not, for each QOL domain, unadjusted for treatment arm nor KS visceral status.
